# A narrative review of routine haematological and biochemical parameter monitoring in maintenance haemodialysis patients and comparison of clinical guidelines

**DOI:** 10.1186/s12882-025-04657-4

**Published:** 2026-01-02

**Authors:** Harry H. Luu, Jade Ryan, Nigel D. Toussaint

**Affiliations:** 1 Department of Nephrology, The Royal Melbourne Hospital (RMH), Grattan Street, Parkville, Victoria, 3052 Australia; 2 School of Medicine and Dentistry, Griffith University, Gold Coast, Australia; 3 Department of Medicine (RMH), University of Melbourne, Parkville, Australia

**Keywords:** Kidney failure, Maintenance haemodialysis, Laboratory testing, Monitoring frequency, Clinical guidelines

## Abstract

Routine monitoring of haematological and biochemical parameters is essential in managing people with kidney failure on maintenance haemodialysis (HD), guiding assessment of HD adequacy and kidney failure-associated complications, including anaemia, chronic kidney disease-mineral and bone disorder, and electrolyte disturbances. Currently, however, there is no consensus on optimal testing frequency of key parameters, with variations across clinical guidelines and limited robust evidence. This narrative review synthesises current guideline recommendations and empirical research on the frequency and scope of routine laboratory testing of people on maintenance HD, focusing on areas of consensus, controversy, and future research needs. Key laboratory parameters were established to standardise the evaluation of foundational and region-specific guidelines, and direct literature searches for research within the last decade. Given the paucity of randomised controlled trials (RCTs), this narrative review is derived primarily from observational research and clinical guidelines. Guideline recommendations varied from flexible, clinician-driven frequencies in foundational guidelines, to prescriptive schedules in region-specific guidelines, though with consistencies across specific parameters. Empirical evidence indicated benefits of reduced testing frequency broadly when addressing medical over-testing. However, limited evidence exists to link parameter-specific testing intervals with outcomes such as hospitalisation, sustaining international variation in guidelines. Future research should pair patient-reported outcome measures with parameter target levels longitudinally in pragmatic RCTs to clarify causal links between optimal monitoring intervals and patient outcomes to develop risk-stratified monitoring frameworks for sustainable HD care.

**Supplementary Information:**

The online version contains supplementary material available at 10.1186/s12882-025-04657-4.

## Introduction

Haemodialysis (HD) is the most common kidney replacement therapy for people with kidney failure. Clinical goals for people with kidney failure focus on improving quality of life; and routine laboratory testing of haematological and biochemical parameters has become integrated into those receiving HD [1]. With recommendations initially outlined by the National Kidney Foundation Kidney Disease Outcomes Quality Initiative (KDOQI) in 1997, establishing appropriate routine pathology testing is essential for assessing dialysis adequacy and preventing and/or managing kidney failure-associated complications such as anaemia, electrolyte abnormalities, and chronic kidney disease-mineral and bone disorder (CKD-MBD) [2].

Frequency of pathology testing, however, must balance a tenuous dichotomy - monitoring to detect modifiable abnormalities, such as iron deficiency, while avoiding over-testing that could yield spurious results, thus escalating unnecessary testing and patient anxiety [1]. In addition, with the rising costs of healthcare, there is increased awareness among clinicians and health services to rationalise the indication for testing in the context of patients’ goals and preferences [3]. Despite advancements in clinical research of HD and the associated sequelae, there is minimal evidence supporting a standardised protocol for pathology monitoring, with variation between international clinical guidelines [1, 3, 4].

The objective of this narrative review was to synthesise current clinical guideline recommendations and empirical evidence for monitoring frequency of key laboratory parameters in patients undertaking in-centre and satellite maintenance HD. By analysing trends in consensus and controversy, and gaps in evidence, focus areas can be established to guide future clinical research in the optimal routine monitoring of pathology tests and frequencies.

## Methods

Two complementary literature search processes were conducted: one targeting clinical guidelines and the other focusing on empirical evidence. International clinical practice guidelines were accessed online to extract recommendations related to routine pathology testing in maintenance HD. Empirical studies were collected through a structured literature search in MEDLINE and Embase (via. Ovid) using the following Medical Subject Headings (MeSH) terms with equivalent keywords: “Renal Dialysis”, “Clinical Laboratory Techniques”, “testing intervals”, and “Health Care Quality, Access, and Evaluation”. Limits were applied to human studies, adults (≥18 years old), and publications from 2000 to 2025 to align with the contemporary focus of this review. The search yielded 283 results after applying limits (Supplementary Table 1 and Supplementary Figure 1).

Subsequently, a two-stage screening process was undertaken to determine alignment with this narrative review’s scope. Studies were included if (i) either specific haematological and/or biochemical parameters, or laboratory testing more broadly, were discussed; (ii) testing frequency, clinical outcomes, and/or effects on healthcare quality or access were evaluated; and (iii) adult patients receiving in-centre maintenance HD were examined.

Exclusion criteria included studies that focussed on modes of dialysis other than in-centre maintenance HD, such as peritoneal dialysis or acute dialysis, or did not have any primary data. In addition, reference lists of included studies were manually searched to identify additional relevant literature. All the sources included were synthesised narratively with attention to key findings, quality, and clinical relevance to contemporary HD.

### Parameters of interest and exclusions

The panel of haematological and biochemical parameters tested are critical for guiding treatment and preventing complications in maintenance HD patients. Laboratory tests of interest for this review include:

- **Haemoglobin (Hb):** Hb is used to monitor for anaemia, a common complication of chronic kidney disease (CKD) especially in patients with kidney failure. Optimisation of Hb within range will inform treatment with erythropoiesis-stimulating agents (ESAs) and iron supplementation, while minimising ESA-related adverse events and cardiovascular risk [1, 4].
- **Iron studies (ferritin and transferrin saturation [TSAT]):** Evaluating iron stores and bioavailability is used to support anaemia management, thereby preventing iron deficiency and overload, while optimising iron therapy and ESA responsiveness [5].
- **Serum electrolytes (potassium [K**^**+**^**], sodium [Na**^**+**^**], bicarbonate [HCO3]):** Patients with kidney failure are disproportionately affected by complications of electrolyte disturbances, especially hyperkalaemia and metabolic acidosis, including life-threatening arrythmias, neuromuscular disturbances, and fluid balance issues [6].
- **Calcium, phosphate, parathyroid hormone (PTH), and alkaline phosphatase (ALP):** Markers of abnormalities in mineral metabolism are monitored to prevent complications associated with CKD-MBD, including valvular and soft tissue calcification, skeletal and bone disease, and cardiovascular morbidity and mortality [7].
- **Albumin and total protein:** General markers of nutritional status and morbidity predictors, with low levels associated with malnutrition, inflammation, and increased risk of adverse events [6].

The following parameters, although considered routine for monitoring of maintenance HD patients for many dialysis units, are not included in this review as analysis of each fall outside the scope of the review and/or parameters have limited research to date on appropriate frequency of testing:

- Infection prevention serology including hepatitis B, hepatitis C, and human immunodeficiency virus (HIV).
- Measures associated with metabolic syndrome including HbA1c, blood glucose levels, and lipid levels.
- Dialysis adequacy measures including Kt/V and urea reduction ratios (URR).

In addition, this review will focus on monitoring pathology testing for in-centre (or satellite) maintenance HD patients, in contrast to people who may be receiving home HD and peritoneal dialysis. In-centre HD may include conventional HD (4–5 hours, thrice weekly), incremental HD (twice weekly), and longer-hours and/or more frequent HD (for example, quotidian, short-daily or nocturnal HD).

Also, timing during the week of routine pre-dialysis pathology testing in maintenance HD is debatable, with mid-week often recommended as this is associated with more stable clinical parameters, and potentially ESA and iron doses, compared to early-week testing. Mid-week measurements may also reflect a more stable volume status, as high interdialytic weight gain and volume fluctuations are typically higher at the beginning of the week after a longer interdialytic gap. In contrast, some would argue routine pathology at the beginning of the dialysis week may be preferred, following the long interdialytic interval usually over the weekend, as this provides worst-case scenario for electrolyte disturbances, but practices vary internationally and there is a lack of high-quality evidence for guidance on practice.

## Discussion

Frequency of routine monitoring of pathology tests is an evolving focus within maintenance HD. The specificity of recommendations for routine pathology testing, both in terms of frequency and parameters included, vary across international clinical guidelines. These discrepancies are multi-faceted, ranging from complex medical comorbidities of the typical patient demographic requiring HD, to systems-level restraints that complicate adherence to the targeted nature of clinical guidelines [3, 8].

One of these systems-level restraints is the principle of medical over-testing, which is driven by a complex interplay of issues including time restraints limiting the development of refined differentials, to notions such as ‘defensive medicine’, whereby healthcare practitioners over-investigate to satisfy patients’ desire for a diagnosis [9–11]. Given that laboratory testing affects up to 70% of treatment decisions, rationalising the appropriateness of testing is essential [12]. Over-testing creates not only unnecessary harm to patients, through increased anxiety and unnecessary testing, but also avoidable expenditure at a health system level and an individual level (e.g., travel expenses, time off work) [13, 14]. Nonetheless, the issues of inappropriate test utilisation must be balanced against the risks of late detection of disease-related complications and the sequelae of clinical deterioration [15]. It is a tenuous balance that continues to be discussed internationally, which is reflected in the current nature of clinical guidelines and emerging research addressing maintenance HD.

### Current nature of clinical guidelines

There is not an internationally accepted protocol for routine pathology testing of people on HD. In the context of varied health needs and challenges for people on HD, difficulties arise when attempting to create clinical guidelines with international applicability. As a result, clinical guidelines have emerged in both local and global settings to inform monitoring principles.

Foundational guidelines, such as those developed from Kidney Disease Improving Global Outcomes (KDIGO), are fundamentally designed to target broad treatment demographics by synthesising international evidence and expert opinion to cover the clinical continuum from initial diagnosis to monitoring and treatment [16]. While providing broad applicability for key clinical principles, these guidelines tend to lack contextual specificity on variations in patient demographics, care delivery models, and resource availability that would influence HD treatment principles. This creates a possibility for recommendations that are impractical, from a resource aspect, or inaccurate, given the slow update cycles inherent to large-scale review structures.

In contrast, region-specific clinical guidelines, often developed by local nephrology societies such as the Japanese Society for Dialysis Therapy (JSDT), adapt foundational principles to local health epidemiology and regulatory considerations. This creates guidelines that are more sensitive to health system priorities, feasibility factors, and local patient demographics that influence therapies such as HD [17]. For example, in JSDT guidelines, Japan’s lower average body weight, compared to Western countries such as the United States and many in Europe, led to variations in Hb cut-off values when compared to international guidelines [18]. Another example is Caring for Australians and New Zealanders with Kidney Impairment (CARI) guidelines, which are showing growing prioritisation of culturally safe care models in-line with national health goals for First Nations Australians [19].

While foundational guidelines are inherently limited to address cultural factors specifically, region-specific guidelines may perpetuate practice principles that are not robustly validated due to the lack of regional data available [20]. However, both foundational and region-specific guidelines are essential resources in providing care for people with kidney failure.

### Monitoring recommendations in clinical guidelines

Despite the common goal to optimise monitoring of people on maintenance HD, recommendations regarding frequency, or type, of testing are not consistent across both foundational and region-specific guidelines. The clinical guidelines included have been summarised in Table 1.

**Table 1:** Summary table of clinical guidelines involving pathology testing of patients on maintenance HD

Clinical Guideline	Year and Geographic Scope	Focus of the Guideline	Key Recommendations and Relevance	Strengths and Limitations in Relation to this Review
**KDOQI:** HD Adequacy [17]	2015 United States (though used internationally with local adaptation).	HD adequacy through (i) optimising dialysis care (incl. timing, frequency, preparation) and (ii) measurement methods for dialysis adequacy.	Outlines baseline standard for monitoring urea-related parameters (*outside scope of this review*). Establishes need for individualised pathology monitoring appropriate to clinical circumstances (e.g., recent hospitalisation requires more stringent monitoring).	**Strengths:** patient-centred care guideline that emphasises the heterogenous nature of clinical care. Refers clinicians to additional/specific guidelines as needed. **Limitations:** no specific biochemical/haematological parameters discussed.
**KDOQI:** Nutrition in CKD [21]	2020 United States (though used internationally with local adaptation).	Recommendations for systematic nutrition assessment and medical nutrition therapy across all stages of CKD to manage metabolic abnormalities.	**Albumin:** outlines importance of albumin as prognostic factor for nutrition status and morbidity; however, no specific monitoring frequency provided.	**Strengths:** transparent evidence grading with rigorous development process. **Limitations:** limited prescriptive guidance on tests, with clear gaps in evidence.
**KDIGO:** Anaemia Management [22]	2025 (note: under public review at time of publication) International	Evidence-based and practical recommendations for management of anaemia in CKD/kidney failure, contextualising pathology monitoring and interventions.	**Hb**: monthly testing in HD patients, inc. to 2-weekly if receiving ESA treatment. **Iron studies**: monthly, if ESA/IV iron-dependent. Provides detailed + actionable protocols for standard + complications-driven monitoring (e.g., onset of new symptoms of unknown cause).	**Strengths:** step-wise algorithm for diagnostic, monitoring, and management of anaemia with global applicability. **Limitations:** ‘optimal frequencies’ defined do not account for practical/adherence/logistical factors.
**KDIGO:** CKD [23]	2024 International	Rigorous evidence base for the evaluation and management of CKD across the entire clinical spectrum, including disease, age, aetiology, and care settings.	**Potassium:** outlines instances to test potassium more frequently, with no monitoring frequencies specifically outlined for HD patients. **General Monitoring:** risk-based assessment and change-triggered tailoring to frequency of testing.	**Strengths:** multi-disciplinary and patient-inclusive framework with methodological rigour and clear practice points for monitoring variability. **Limitations:** limited scope of inclusion for people on maintenance HD
**KDIGO:** CKD-MBD [20]	2017 International	Monitoring, diagnosis, and prevention of CKD-MBD, with treatment guidelines for abnormalities in calcium, phosphate, and PTH.	**Calcium + Phosphate =** 1–3 monthly. **PTH =** 3–6 monthly. **ALP =** annually (more if elevated PTH). Emphasises trends-based analysis to influence changes in frequency (i.e., not prescriptive).	**Strengths:** broad guideline from prevention to management. **Limitations:** lack of robust RCTs/high-quality evidence, with guidelines based on expert consensus.
**KSN**: Optimal HD [7]	2021 Republic of Korea	Provide standardised clinical practice guidelines for people in Korea receiving HD to improve health outcomes and quality of life.	**Monthly:** full blood count (incl. Hb); liver function tests; urea, electrolytes, & creatinine; calcium + phosphate. **3-monthly:** iron studies, PTH, HbA1c, chest x-ray. **6-monthly:** hepatitis viral markers + ECG Draws from international best practice (KDIGO, KDOQI, European Renal Association) to create guidelines for medical landscape in the Republic of Korea.	**Strengths:** transparent and methodological approach to recommended testing intervals, specifically referencing where international guidelines were incorporated. **Limitations:** specific frequencies do not have high quality evidence (i.e., based on KSNs consensus on appropriate frequency), particularly for investigations not commonly conducted (e.g., chest x-ray, ECG).
**JSDT:** HD [24]	2015 Japan	Outline minimum standards for long-term, stable maintenance HD to improve survival and quality of life.	No outline of specific biochemical and haematological parameters. Emphasises requirement for close monitoring of sequelae of CKD/kidney failure (e.g., anaemia, CKD-MBD) as per international guidelines, with regular nutritional assessments due to local health needs (i.e., higher elderly population, smaller body size).	**Strengths:** recommendations are supplemented with grading system of quality of evidence available. **Limitations:** parameters are not explicitly included (e.g., monitor for signs of CKD-MBD vs. calcium/phosphate specifically).
**JSDT:** Anaemia [18]	2015 Japan	Provide guidance on diagnosis, monitoring, and individualisation of treatment of anaemia in CKD/kidney failure.	**Hb, haematocrit:** recommended routine monitoring and reference ranges; however, no frequency provided. **Iron studies:** 3-monthly if on ESA-therapy. Encourages assessment of complications (e.g., blood pressure, symptoms, treatment hypo-responsiveness) to titrate monitoring, with no specific recommendations on how to change frequency.	**Strengths:** comprehensive, locally-adapted standard-of-care reference for people across spectrum of CKD **Limitations:** lack of explicit monitoring frequencies with generality of recommendations, emphasising use of clinicians’ judgement to guide frequency of testing.
**CARI:** Hb [25]	2012 Australia and New Zealand	Provides Hb targets and monitoring practices to alleviate anaemia and polycythaemia-related symptoms, while reducing need to adjust ESA dose ± require iron infusions.	**Hb:** at least monthly and after ESA dose changes, with rises not exceeding 10 g/L per two-week period. **Iron Studies:** 3-monthly. Also outlines additional assessments if Hb rise is not sufficient.	**Strengths:** specific practical guidelines that incorporate risk-benefit analysis, with strong emphasis on expected trends in parameters. **Limitations:** gaps in evidence for long-term outcomes and quality of life.
**CARI:** Iron [26]	2013 Australia and New Zealand	Optimise iron therapy in people with CKD/kidney failure and anaemia and minimise risks associated with iron deficiency and excessive supplementation.	**Hb:** every 1–3 months if stable; 2–4 weekly during correctional phase. **Iron Studies:** every 1–3 months (moreso circumstantially – ESA dose change, blood loss, after IV iron infusion). Encourages dynamic adjustments while contextualising to factors including clinical status, comorbidities, intensity of HD treatment, and limitations to testing.	**Strengths:** comprehensive guide on anaemia and iron deficiency management while recognising gaps in evidence. **Limitations:** heterogenous evidence with dose-response, outcome monitoring, and patient-centred endpoints, limiting generalisability.
**UKKA:** HD [27]	2019 United Kingdom (though used internationally with local adaptation).	Outline standards for “good quality HD” in terms of patient safety & experience, good clinical practice, and holistic, patient-centred dialysis practice.	**Electrolytes:** details specific biochemical ranges for potassium (4-6 mmol/L), and bicarbonate (18-26 mmol/L), and cross-references UKKAs hyperkalaemia guideline for monitoring frequency [28]. **Albumin/Total Protein:** no frequency listed; mentions albumin + total protein as markers of nutrition, in-line with other clinical guidelines.	**Strengths:** methodological approach to assessing evidence strength, with multidisciplinary authors. **Limitations:** minimal prescriptive laboratory testing advice, with heterogenous evidence base.
**UKKA:** Anaemia [29]	2025 United Kingdom (though used internationally with local adaptation).	Provide an updated, evidence-based recommendation for the management of anaemia in CKD/kidney failure, endorsing alternative guidelines as relevant.	**Hb:** at least monthly testing, with key baseline laboratory investigations in newly diagnosed cases of anaemia. Outlines aim to maintain Hb > 110 g/L. **Iron Studies:** outlines testing one- to three-monthly in patients on iron therapy; however, no specific recommendation on those not on ESA/IV iron.	**Strengths:** contemporary and comprehensive update that assesses quality of evidence and cross-references other major guidelines. **Limitations:** doesn’t address specific iron studies in people not on ESA/IV, with evidence gaps in testing frequency.
**UKKA:** CKD-MBD [30]	2018 United Kingdom (though used internationally with local adaptation).	Provide commentary on the 2017 KDIGO update, focusing on the notion of adopting pragmatic, individualised, and multidisciplinary care in monitoring biomarker trends.	**Calcium, phosphate, PTH:** no explicit frequencies are recommended, rather emphasising importance of serial measurements/trends over single absolute values tied into clinical context and treatment decisions.	**Strengths:** provides updated risk perspective to account for harms/benefits of testing principles proposed. Great emphasis on multidisciplinary input with audit metrics. **Limitations:** lack of prescriptive testing intervals with substantial gaps in evidence.

**Abbreviations**: ALP, alkaline phosphatase; CARI, Caring for Australians and New Zealanders with Kidney Impairment; CKD, chronic kidney disease; CKD-MBD, chronic kidney disease – mineral bone disorder; ECG, electrocardiogram; ESA, erythropoiesis-stimulating agent; Hb, haemoglobin; HD, haemodialysis; IV, intravenous; JSDT, Japanese Society for Dialysis Therapy; KDIGO, Kidney Disease Improving Global Outcomes; KDOQI, Kidney Disease Outcomes Quality Initiative; KSN, Korean Society of Nephrology; IV, intravenous; PTH, parathyroid hormone; RCTs, randomised controlled trials; UKKA, UK Kidney Association

#### Prescriptive vs. Suggestive guidelines on frequency of pathology monitoring

A dichotomous quality is evident in the tone of recommendations. Specifically, a contrast in suggestive tone where either clinicians are encouraged to adapt guideline recommendations or prescriptive language that emphasises the protocolisation of standardised care principles. Foundational guidelines tend to adopt suggestive language that encourage individual interpretation by clinicians referring to these guidelines. Phrases such as “it seems reasonable to” and timespans such as “every 2–4 weeks to monitor Hb” endorse clinical judgement to supplement the recommendations outlined [18, 22]. Conversely, some region-specific guidelines, particularly those by JSDT and KSN, favour prescriptive language that aims to standardise care, such as outlining specific parameters to test: “we recommend complete blood count (CBC), liver function, routine blood chemistry …” [7]. While reflecting a goal for system-wide standardisation and quality benchmarking, this protocol-driven language de-emphasises clinical judgement for changing monitoring frequencies. Though guidelines are not restricted by these trends, this feature is important to acknowledge when analysing variations in routine pathology testing recommendations.

#### Haemoglobin

Monitoring of Hb is often tested under a Full Blood Count (FBC) or CBC. While a standard parameter to monitor in all people on HD, variations in frequency are often due to complications related to CKD-anaemia or ESA treatment.

- **KDIGO:** recommends monthly testing of Hb in people undergoing HD, increasing up to fortnightly if unstable anaemia management or starting/changing doses of ESA-therapy [22].
- **KSN:** recommends monthly testing of Hb in people undergoing HD; however, no specific recommends for those with known anaemia or ESA therapy. Directly quotes KDIGO as reference point for these specific considerations [7].
- **JSDT:** no frequency is recommended; instead, outlines target Hb range of 10 to 11 g/dL, encouraging clinicians to adjust ESA doses (if on treatment) and/or monitoring until Hb levels normalise. JSDT comment on “European and US” guidelines for Hb as irrelevant to the Japanese population, due to lower average dry weights, hence a deviation in the standard of recommendations [18].
- **CARI:** recommends at least monthly and/or one month after ESA dosage change, aligning with KDIGO. In addition, outlines a Hb target between 100 to 115 g/L, with rises not exceeding 10 g/L per two weeks [25].
- **UK Kidney Association (UKKA):** outlines at least monthly testing with a Hb target > 110 g/L, contrasting ranges provided by other clinical guidelines [29]

Of note, CARI outlines that their guidelines for Hb monitoring do not account for people who are ESA-naïve, reflecting a potential indication for less frequent monitoring in people undergoing HD who do not have anaemia, or are stable [25]. This is seen by KDIGO’s recommendation for three-monthly testing in people on HD without anaemia. This is also acknowledged by KSN, who outline emerging research into reduced frequency of monitoring; however, continuing to recommend monthly testing in accordance with standard practice [7].

#### Iron studies (ferritin, transferrin saturation [TSAT])

Similar to Hb, monitoring an individual’s iron studies often depends on the presence of CKD-anaemia and/or ESA therapy.

- **KDIGO:** individuals who do not receive ESA therapy, have a history of iron-deficiency, or CKD-anaemia should be tested every three months. However, this increases to monthly in patients who are ESA or intravenous (IV) iron dependent [22]. KDIGO’s 2025 anaemia guideline was under public review at the time this manuscript was published.
- **KSN:** iron status testing should be conducted *at least* every three months, without clear adjustments for those on ESA therapy or iron supplementation, based on expert consensus [7].
- **JSDT:** iron status should be assessed *at least* every three months, with more frequent examinations when starting ESA therapy – though no specific timeframe is provided [18].
- **CARI:** regular monitoring of iron status is required for people on HD, with testing recommended every one to three months. No specific adjustments are recommended for those on ESA therapy [26].
- **UKKA:** recommends testing every one to three months if people are on ESA or IV iron therapy; however, no specific recommendations are outlined for patients who do not require these therapies. UKKA acknowledge that most people on HD will require IV iron [29].

Testing individuals’ iron status remains consistent across all major clinical guidelines; however, adaptive testing based on concurrent Hb levels and potential ESA hypo-responsiveness remains varied when comparing guidelines. For example, KDIGO explicitly acknowledge dose changes of ESAs, known blood loss, hospitalisation, or abnormal trends in TSAT or ferritin as indications to increase testing frequency [22]. Conversely, less frequent testing recommended by KSN was attributed to the “medical reality” and “cost of testing”, though not dismissing increased testing if required [7]. This contrasts reasoning outlined by JSDT guidelines, which outline potential risks of initiating iron replacement therapy prematurely due to over-monitoring; however, the guidelines acknowledge the benefit of more frequent iron status testing for those with established iron deficiency or ESA therapy [18].

#### Serum electrolytes

Potassium, sodium, and bicarbonate are common serum electrolytes monitored in maintenance HD due to disproportionate rates of electrolyte disturbance in people with kidney failure. However, compared to the previous parameters, specificity and emphasis on electrolyte monitoring varies.

- **KDOQI:** hyperkalaemia, volume control/sodium levels, and metabolic acidosis (thus indirectly, bicarbonate) are listed as factors to assess adequacy of an individual’s HD regimen. However, specific frequency intervals are not provided [17].
- **KDIGO:** the 2024 CKD guidelines, though not overly specific to people on maintenance HD, briefly outlines the importance of individualised management of hyperkalaemia in people with CKD 3-5. Although there are additional recommendations on instances to test potassium more frequently (e.g., 2-4 weeks after initiating an angiotensin-converting enzyme inhibitor [ACEi] or angiotensin receptor blocker [ARB]), no monitoring frequencies are specifically outlined for people on maintenance HD [23].
- **KSN:** potassium and sodium are explicitly listed as ‘routine blood chemistry’ for at least monthly monitoring testing, with no specific schedule for bicarbonate testing [7].
- **JSDT:** similar to KDOQI, potassium and sodium are referenced indirectly through monitoring for hypokalaemia and volume control, respectively. However, no specific frequency intervals are provided [18].
- **UKKA:** three to six monthly testing of potassium is recommended; no testing frequencies are provided for other electrolytes [28]. Pre-dialysis levels for potassium (4-6 mmol/L) and bicarbonate (18-26 mmol/L) are outlined [27].

#### Calcium, phosphate, PTH, and ALP

These parameters are commonly monitored in the context of CKD-MBD and associated sequelae, generally with consistent monitoring intervals across clinical guidelines.

- **KDIGO:** serum calcium and phosphate tested every 1-3 months; PTH every 3-6 months; and ALP annually. KDIGO also suggests measuring vitamin D in known deficiency, or if interventions may affect levels [20]. KDOQI and CARI defer to KDIGO’s CKD-MBD guideline for relevant monitoring intervals [17].
- **KSN:** serum calcium and phosphate tested at least monthly; and PTH at least 3-monthly. [7]. Compared to KDIGO, more stringent intervals are provided using the lower limits of KDIGO’s recommendations. ALP is not explicitly recommended.
- **JSDT:** do not provide explicit testing intervals; however, the guideline outlines complications associated with hypocalcaemia and hyperphosphatemia, and the consequential bone mass reduction, with poor monitoring [24].
- **UKKA:** no testing frequencies are provided despite commentating on KDIGO’s CKD-MBD guidelines; instead, UKKA emphasises the importance of serial measurements of calcium, phosphate, and PTH and developing individualised ranges for people on HD [30].

Despite variations in recommended frequencies, there is a clear consensus across all guidelines to assess CKD-MBD-associated parameters based on trends, rather than single laboratory values, and to adjust frequency of monitoring proactively when evaluating treatment efficacy (e.g., phosphate binders, calcimimetics) or magnitude of abnormalities.

#### Albumin and total protein

As markers of nutritional and morbidity status, monitoring recommendations remain limited across guidelines.

- **KDOQI:** cross-referenced by KDOQI’s Nutrition in CKD 2020 Guideline, albumin and total protein are outlined as key parameters in assessing nutrition status, risk of hospitalisation, and mortality risk of people on maintenance HD [21]. However, no explicit monitoring frequency is provided. Similar recommendations outlined by KDIGO, CARI, and UKKA [21, 27].
- **KSN:** testing of total protein and albumin is recommended at least monthly in conjunction with liver function tests [7].
- **JSDT:** states that serum albumin should be measured “regularly”, citing potential for large amounts of albumin loss in HD and associated complications (e.g., oedema) – an alternative rationale compared to other clinical guidelines [24].

#### Concluding trends in clinical guidelines

Clinical guideline recommendations remain reliant on expert-informed consensus, rather than high-quality evidence, due to the limited coverage of RCTs in nephrology [31, 32]. The heterogeneity of clinical guideline recommendations (outlined in Table 2) present risks for both over-testing, such as associated patient-level and systems-level costs, and under-testing, which can lead to missed clinical deterioration and compromised patient outcomes. This underscores the importance of high-fidelity research to substantiate clinical guideline recommendations, especially given the fundamental role of clinical guidelines supporting healthcare practitioners in circumstances of clinical decision-making uncertainty.

**Table 2:** Clinical guideline recommendations for monitoring frequency of haematological and biochemical parameters in HD

	Haemoglobin	Iron Studies (Ferritin, TSAT)	Serum Electrolytes (Potassium, Sodium, Bicarbonate)	Calcium, Phosphate, PTH, and ALP	Albumin and Total Protein
**KDOQI**	N/A.	N/A.	**No frequency**: monitor for hyperkalaemia, volume control (Na^+^), and metabolic acidosis (HCO_3_) [17].	*Defer to KDIGO*.	**No frequency:** outline albumin as key parameter in nutrition status and morbidity risk calculation [21].
**KDIGO**	**Monthly** (fortnightly if unstable) [22].	**Three-monthly.** **Monthly** if on ESA or IV Iron Therapy [22].	**No frequency:** individualised monitoring, particularly after new change (e.g., starting ACEi/ARB) [23].	**One to three monthly:** calcium & phosphate. **Three to six monthly:** PTH. **Annually:** ALP [20].	**No frequency:** outline albumin as key parameter in nutrition status and morbidity risk calculation [21].
**KSN**	**Monthly.**	**Three-monthly**.	**At least monthly:** K^+^ and Na^+^ (HCO_3_ not mentioned).	**At Least monthly:** calcium & phosphate. **At Least 3 monthly:** PTH. ALP not mentioned.	**At least monthly.**
**JSDT**	**No frequency:** aim Hb 100–110 g/L [18].	**Three-monthly**, more frequent if on ESA *(no specific value provided* [18].	**No frequency:** monitor for hyperkalaemia and volume control (Na^+^). HCO_3_ not mentioned [24].	**No frequency:** advise to monitor for hypocalcaemia and hypophosphate-mia, and consequential bone loss [24].	**No frequency:** recommends to test serum albumin “regularly” [24].
**CARI**	**No frequency:** aim Hb 100–115 g/L [25].	**One to three monthly** [26].	N/A.	*Defer to KDIGO.*	**No frequency:** outline albumin as key parameter in nutrition status and morbidity risk [21].
**UKKA**	**Monthly** (as per KDIGO); aim Hb > 110 g/L [29].	**One to three monthly** if on IV Iron Therapy [29].	**Three to six monthly:** monitor K^+^ in patients with diabetes, heart failure [28]. Pre-dialysis K^+^ and HCO_3_ values provided [27].	**No frequency:** commentate on KDIGOs guidelines, suggesting serial measurement of calcium, phosphate, and PTH to individualise targets [30].	**No frequency:** outline albumin and protein as a marker of nutrition [27].

**Abbreviations:** ACEi, angiotensin-converting enzyme inhibitor; ALP, alkaline phosphatase; ARB, angiotensin receptor blocker; CARI, Caring for Australasians with Kidney Disease; CKD-MBD, chronic kidney disease – mineral bone disorder; ECG, electrocardiogram; ESA, erythropoiesis-stimulating agent; Hb, haemoglobin; HCO_3_, bicarbonate; HD, haemodialysis; IV, intravenous; JSDT, Japanese Society for Dialysis Therapy; K^+^, potassium; KDIGO, Kidney Disease: Improving Global Outcomes; KDOQI, Kidney Disease Outcomes Quality Initiative; KSN, Korean Society of Nephrology; Na^+^, sodium; PTH, parathyroid hormone; TSAT, transferrin saturation; UKKA, United Kingdom Kidney Association

### Current research into frequency of laboratory testing

Routine laboratory testing is an integral component in the care of people on maintenance HD, with testing frequency becoming a growing area of research interest. Although protocolised monitoring of haematological and biochemical parameters support early detection of clinical deterioration, as emphasised by Kim et al. (2023), evidence on optimal monitoring intervals remains limited, presenting challenges for patient safety, outcome optimisation, and resource utilisation [6].

Current evidence available is primarily observational and retrospective in design, with studies also varying in scope – ranging from broad laboratory testing panels, to specific parameters (Table 3). Overall, these clinical studies evaluated whether less frequent testing could maintain patient outcomes, including patient-reported outcome measures (PROMs), without compromising care.

**Table 3:** Studies assessing different frequencies of routine pathology testing in patients on maintenance HD

Study	Participants and Setting	Brief Study Description	Intervention	Key Findings and Relevance to Review	Study Design Critical Appraisal
Kim et al. (2023) [6]	34,590 adult patients undergoing HD ≥2x/wk at 798 facilities throughout Korea.	Longitudinal observational cohort study conducted in 2015, with 85.6% of participants undergoing regular pathology testing.	Does not explore optimal frequency, rather examined testing (85.6% of cohort) vs. non-regular testing (14.4% of cohort).	**Prim. Outcome:** lower all-cause mortality (HR 0.9, 95% CI 0.85–95) and crude mortality rate (79 vs. 85 per 1,000 person-years) with testing. **Sec. Outcome(s):** lower serum phosphate and diastolic BP; and higher Hb and dialysis adequacy with testing. **Relevance:** supports regular pathology testing and importance of protocolised pathology monitoring of parameters.	**Strengths:** large, national sample size with long follow-up (avg. = 53.7 months); sub-group analysis conducted for confounding variables (e.g., diabetes, IHD). **Limitations:** lack of data on therapeutic interventions (e.g., how therapy was influenced by laboratory testing); occurrence of comorbidities and cost-effectiveness not measured. **Generalisability:** good internal/national generalisability, with potential international applicability given health system differences in Korea.
Thomas et al. (2020) [1]	18,120 HD patients receiving blood tests in patient cohorts in Ontario, Canad from April 1 2011 to March 31 2016.	Retrospective population-based cohort study.	Examine four-weekly (*n* = 13,087) vs. 6-weekly (*n* = 5,033) surveillance blood work and effects on clinical outcomes	**Prim. Outcome:** four-weekly testing was not associated with lower all-cause mortality (collected from Registered Persons Database) compared to 6-weekly. **Sec. Outcome(s):** in four-weekly testing, there was a higher risk of CVS events (HR 1.18), hospitalisation (HR 1.11), and hyperkalaemia (HR 1.20). **Relevance:** challenges the notion that more frequent (four-weekly) testing is beneficial, emphasising risks of over-testing.	**Strengths:** province-wide, population-based design that focuses on clinical outcomes (e.g., mortality, CVS events, hospitalisations) over surrogate markers. **Limitations:** observational, non-randomised design leads to unmeasured confounding; does not measure/control additional or unscheduled blood tests outside of routine intervals. **Generalisability:** limited applicability to paediatric populations and/or regions with limited access to publicly funded dialysis or laboratory testing.
Chidiac et al. (2022) [5]	210 patients undergoing HD for > 12 months at three centres in Lebanon.	Retrospective observational cohort study using data from Feb 2012 to Feb 2011.	Examine groups of patients that would benefit from increased or decreased laboratory blood testing by examining intervention-to-testing ratio.	**Prim. Outcome:** stable HD patients would benefit from less frequent, with two-monthly Hb + calcium + phosphate testing; and 6-monthly PTH testing. **Sec. Outcome:** people with anaemia management, smokers, high-trending PTH/phosphate, and started dialysis at a younger age require more frequent testing. **Relevance:** advocates for nuanced, patient-specific monitoring protocols in HD.	**Strengths:** multi-centre design with clinically actionable metrics (e.g., median number of yearly prescription changes) to inform testing interval proposals + cost-saving considerations. **Limitations:** potential selection bias as people on dialysis < 12 months excluded (favours more stable patients); retrospective design. **Generalisability:** study includes typical HD comorbidities and demographics (i.e., age, diabetes).
Silver et al. (2019) [4]	HD patients, approximately 350 to 400 annually, receiving treatment at a tertiary hospital in Ontario, Canada.	Retrospective interrupted time series from June 1 2012 to December 31 2015.	Determine effect of changing frequency of routine laboratory testing from four-weekly (697 person-years) to six-weekly (766 person-years) on achieving CKD-MBD and anaemia targets (as per KDIGO).	**Prim. Outcome:** Hb (anaemia) and phosphate (CKD-MBD) targets remained stable at average 60 and 46%, respectively, after switching from four-weekly to 6-weekly testing. **Sec. Outcome:** calcium and PTH targets did not exceed limit with reduced testing frequency; saved ~CA$35,000 with six-weekly testing. **Relevance:** clinical targets remained stable with additional health resource benefit with reduced testing.	**Strengths:** robust study design that limited heterogeneity in implementation, with long period of outcomes monitoring (18 months). **Limitations:** absence of case-mix adjustment and data for acute interventions (e.g., hospitalisation) could precipitate individual-level confounding. **Generalisability:** single, tertiary centre so findings may not generalise to centres with different patients or staffing/care models.
Shome-Vasanthan et al. (2024) [33]	972 patients receiving in-centre HD at a health service in Alberta, Canada.	Mixed retrospective control group (October 31 2019 – Oct 31 2020) and prospective intervention group (Oct 31 2020 – Oct 31 2021).	Determine safety and clinical impact of reducing surveillance blood work in HD patients from six-weekly to eight-weekly.	**Prim. Outcome:** successfully reduced testing from 40 to 51 days; anaemia (Hb & TSAT) and CKD-MB (calcium, PTH) markers remained in-target with reduced testing. Phosphate had reduced odds of being within target (OR = 0.91). **Sec. Outcome**: no significant difference in all-cause mortality; hospitalisations reduced with eight-weekly testing. **Relevance**: outlines capacity to reduce testing frequency without sacrificing quality of care in light of healthcare resource and efficiency strains.	**Strengths:** large, prospective design that measures both biochemical & clinical outcomes, adjusting for covariates with mixed + zero-inflated regression models. **Limitations:** temporal confounding as control period predated the COVID-19 pandemic, while intervention group occurred during the pandemic. **Generalisability:** generalisable to adult maintenance in-centre HD, though considerations for hospital practice during the COVID-19 pandemic should be considered.
Gaweda et al. (2010) [34]	49 patients undergoing HD in Massachusetts, United States of America.	Prospective interventional case-controlled trial.	Analyse how sampling frequency affects accuracy and reliability of Hb in monitoring of Hb patients.	**Prim. Outcome:** calculated Hb estimation error, based on device measurement error, and determined 4 Hb tests/month was optimal clinical frequency to minimise errors. **Sec. Outcome:** optimal monitoring frequencies based on patient preference − 45% monthly, 31% twice-weekly, and 12% bi-monthly or monthly. **Relevance:** advanced analytical technique with intensive sampling and unique focus on error minimisation, foregoing system restraints.	**Strengths:** prospective, high-frequency data collection with rigorous quantitative analysis to identify theoretically optimal testing interval. **Limitations:** small single-centre cohort with limited evaluation of practical considerations (i.e., frequency of blood draws). **Generalisability:** limited external validity in relation to logistical or clinical considerations for HD, though provides theoretical basis for optimal testing frequency.
Yokoyama et al. (2017) [35]	3279 patients on HD with secondary hyperparathyroid-ism across multiple centres in Japan from January 2008 to January 2011.	Multi-centre, prospective cohort study over a 3-year follow-up period.	Assess whether increased monitoring frequency of mineral metabolism markers (calcium, phosphate, PTH) improved achievement of target ranges. Calcium + phosphate = weekly, biweekly, monthly PTH = monthly, bimonthly, trimonthly.	**Outcomes:** When outside of range, calcium + phosphate had increased likelihood of reaching target levels with increased/weekly testing (OR = 1.57, 95% CI 1.09–2.26). Increased/monthly testing of PTH had positive effects if outside of range (OR 1.14, 95% CI 1.01–1.27). If values were in-range, increased monitoring was not associated with improved maintenance. **Relevance:** supports intensive episode-based testing when patient’s values are out-of-range.	**Strengths:** large, multi-centre, prospective study with thorough statistical analysis to account for intra-individual & time-varying confounding. **Limitations:** potential variations in true testing frequency (i.e., additional tests) and interventions (e.g., diet advice) could create unaccounted confounding variables. **Generalisability:** use of JSDT guidelines could affect applicability to countries/centres using different guidelines.
Auon et al. (2022) [36]	79 HD patients in Lebanon.	Observational evaluation of cost-saving scheme in a sample of HD patients.	Assess safety and outcomes of a cost-saving HD centre that reduced both frequency of dialysis and laboratory blood testing.	**Prim. Outcome:** reduced dialysis frequency was well tolerated, with only 5/24 of twice-weekly patients suffering adverse events (and shifted back to thrice-weekly HD). **Sec. Outcome:** patients with stable ESA treatment for anaemia did not suffer poorer clinical outcomes with reduced Hb testing. **Relevance:** in resource-scarce areas, judicious reduction in dialysis and testing frequency can be safe in HD patients.	**Strengths:** pragmatic evaluation of acute resource crisis management in HD, with clear outcome data analysis. **Limitations:** small sample & intervention group with short follow-up; sub-optimal analysis of confounder limits statistical power. **Generalisability:** primarily applicable to low-income settings with severe resource constraints.

**Abbreviations:** avg., average; CI, confidence interval; CKD-MBD, chronic kidney disease-mineral bone disorder; CVS, cardiovascular; ESA, erythropoiesis-stimulating agent; Hb, haemoglobin; HD, haemodialysis; HR, hazards ratio; IHD, ischaemic heart disease; JSDT, Japanese Society for Dialysis Therapy; KDIGO, Kidney Disease: Improving Global Outcomes; n, number; OR, odds ratio; Prim., primary; PTH, parathyroid hormone; Sec., secondary; TSAT, transferrin saturation

#### A broad evaluation of less frequent testing

A large population-based Canadian study by Thomas et al. (2020) compared 4-weekly and 6-weekly blood testing, and found that those receiving 4-weekly testing were at a higher risk of cardiovascular event (adjusted hazard ratio [HR] 1.18), all-cause hospitalisation (adjusted HR 1.11), and hyperkalaemia (adjusted HR 1.20), compared to 6-weekly testing [1]. Given there was no statistically significant difference in all-cause mortality between the two groups over the study’s five-year period, these findings were attributed to spurious abnormalities and thus the potential harm of over-monitoring [1]. Similarly, a multicentre study conducted in Lebanon by Chidiac et al. (2022), found that 6-weekly testing did not hinder the quality of patient care, particularly in stable maintenance HD patients [5]. However, this study posited that certain demographics – namely people with anaemia, smokers, and those who started dialysis at a younger age – warranted closer, more frequent monitoring [5]. This presents a more nuanced evaluation of confounding variables that may have been unmeasured by Thomas et al.

Two other studies by Silver et al. (2019) and Shome-Vasanthan et al. (2024) explored switching from a 4-weekly to 6-weekly interval and 6-weekly to 8-weekly interval, respectively, and found no statistically significant difference in all-cause mortality after reducing frequency of routine pathology testing [4, 33].

Taken together, these four studies indicate that modest reductions of routine pathology testing frequency, particularly in stable adults on maintenance HD, did not worsen morbidity or survival and may minimise risk associated with over-monitoring. However, rather than treating laboratory testing as a single entity, it is important to acknowledge the clinical purpose of specific parameters. Therefore, a more nuanced analysis is required to inform risk-adaptive monitoring in maintenance HD.

#### Hb and iron studies in the context of anaemia

Studies into testing frequency in anaemia monitoring have reported varying results depending on focus. Shome-Vasanthan et al. suggested that two-monthly testing would be viable in most patients due to the lack of negative effects on all-cause mortality and hospitalisations with reduced testing frequency [33]. Chidiac et al. showed a low intervention-to-testing ratio after reducing pathology testing to 6-weekly, corroborating with the reduced testing frequency recommendation for Hb, reporting an average intervention-to-testing ratio of 5 ESA dose changes for 12 Hb readings. However, this study purports that people with existing anaemia management derangements, such as low Hb or TSAT, required increased monitoring [5].

A study by Gaweda et al. (2010) used Fourier analysis to determine the optimal Hb frequency testing to minimise incorrect ESA dosing. This study determined that weekly testing balanced error minimisation and was most effective; however, the study was conducted over a short sampling period with a small cohort [34]. Overall, evidence supports individualised testing guided by patient stability, such as increased monitoring when managing unstable anaemia or adjusting ESA dosing.

#### CKD-MBD and mineral metabolism parameters

Both Silver et al. and Shome-Vasanthan et al. found that calcium and PTH remained within target ranges when testing frequency was reduced; however, phosphate appeared more sensitive to interval changes and became more unstable with reduced monitoring [4, 33]. These findings are supported by Yokoyama et al. (2017), which suggested phosphate was the only mineral metabolism parameter that had a decreased likelihood of reaching target levels with less frequent testing [35].

Yokoyama et al. also found that increased monitoring did not improve maintenance of mineral metabolism markers once they were in target levels [35]. Therefore, this study proposes the benefit of intensive episode-based testing compared to default guideline frequencies when parameters are deranged. This notion is supported by Chidiac et al. who found benefit of increased monitoring in patients with high-trending PTH and/or phosphate [5].

#### Electrolyte derangements

Research focussed on frequency of electrolyte monitoring is limited. Thomas et al. identified increased risk of hyperkalaemia with 4-weekly testing, compared to 6-weekly, without increased risk of all-cause mortality, reflecting a transient abnormality without true harm from reduced monitoring [3]. This is supported by the study by Shome-Vasanthan et al., which found an 18% increased risk of hyperkalaemia in 6-weekly testing, compared to 8-weekly testing, with an absence of critical clinical events [33]. Evidence for other essential electrolytes in maintenance HD, including sodium and bicarbonate, is minimal and should be addressed in future research.

### Resource considerations and low-resource settings

Optimisation of healthcare resource allocation is an important consideration for clinical practice, particularly for HD centres managing chronic patients with high weekly treatment loads. A study by Auon et al. (2022) sought to assess the safety of cost-saving HD schemes in resource-scarce clinical settings, which supported pathology testing 6-weekly as a viable cost-reducing change for stable patients [36]. Silver et al. and Shome-Vasanthan et al. found that reducing pathology testing frequency yielded program-wide savings of approximately CA$35,000 and CA$32,962, respectively [4, 33]. However, there is insufficient evidence available to substantiate the most cost-effective testing interval in HD that maintains patient safety while optimising resource utilisation. Of note, rationalising testing is not solely about reducing cost but about redirecting laboratory and personnel resources toward clinically meaningful, patient-centred activities – a broader systems-level view which aligns with current healthcare sustainability frameworks.

### Current consensus and gaps in research

Overall, clinical research has shown that reduced frequency of pathology testing in maintenance HD patients yielded no increased risk of all-cause mortality or significant morbidity. Although guidelines now recommend reduced testing frequency for certain parameters (e.g., PTH), direct evidence linking the testing frequency of specific parameters to outcome measures, such as hospitalisations and dialysis adequacy, remain limited. This fact, coupled with specific clinical scenarios that require more intensive monitoring, such as patients with unstable anaemia, perpetuate the heterogeneity in clinical guideline recommendations internationally. Therefore, there is value in distinguishing mandatory safety-driven monitoring of parameters directly linked to acute clinical risk, such as Hb or potassium, and discretionary optimisation-driven monitoring, such as iron studies. Integrating these nuances will create more intentional and practical frameworks to guide future research and clinical guideline development.

Current research also emphasises the importance of prioritising PROMs, such as symptom burden and quality of life, over surrogate markers and whether laboratory parameters are ‘in-range’. Future studies should operationalise PROMs within trial design, moving beyond descriptive associations, through pragmatic RCTs or prospective research designs that pair longitudinal PROM trajectories with haematological and biochemical trends. Stronger causal inference between testing frequency, health outcomes and patient experience could therefore be examined.

In addition, future cost-effectiveness evaluations should examine the re-allocation of personnel and laboratory resources from routine low-value testing to patient-centred interventions including symptom management and psychosocial support. This research could inform system-wide sustainability efforts in maintenance HD as research into testing frequency becomes more integrated with PROMs.

Comparison between empirical evidence and clinical guidelines highlight a lack of robust data for fundamental laboratory parameters in the monitoring of people on maintenance HD – including sodium, albumin, and ALP. Future research should evaluate the viability of risk-stratified and individualised testing regimens that are guided by PROMs, comorbidities, and therapy changes. These processes can potentially be enabled by clinical support tools such as artificial intelligence-driven algorithms and electronic health record-integrated analytics, which are becoming increasingly integrated into digital health apparatuses [37, 38].

Several limitations of this review should be acknowledged. As a narrative review, the search strategy was non-systematic given the absence of high-fidelity RCTs and grey literature to substantiate search findings. In addition, the reliance on English-language studies and published guidelines may have limited the breadth of reviewed materials.

## Conclusion

There remains a disparity between clinically significant parameters in the care of people on maintenance HD, the degree of specificity regarding testing frequency in clinical guideline recommendations, and the strength of empirical evidence supporting testing frequency. As a result, clinical guideline recommendations remain largely heterogenous and informed by expert consensus rather than robust evidence.

Emerging research indicates that targeted reductions in testing frequency can optimise management without compromising quality of care, if decision-making incorporates patient-centred metrics. While clinical guidelines remain focused on maintaining target levels for parameters, future research requires a nuanced integration of PROMs longitudinally with laboratory parameters using risk-adaptive protocols, particularly when evaluating the necessity of testing and risks associated with over-testing and late detection. To translate future research into sustainable guidelines and protocols, interdisciplinary collaboration between healthcare practitioners and policy-makers is essential to develop a monitoring model that safely balances patient-centred care with clinical precision, thereby optimising outcomes for people on maintenance HD.

## Electronic supplementary material

Below is the link to the electronic supplementary material.

Supplementary Material 1

## Data Availability

No datasets were generated or analysed during the current study.
