# Supplementary material for: A narrative review of routine haematological and biochemical parameter monitoring in maintenance haemodialysis patients and comparison of clinical guidelines
Source: BMC Nephrol. 2026 Jan 2;27:19. doi: 10.1186/s12882-025-04657-4 (PMC12784567; doi:10.1186/s12882-025-04657-4)
Supplement: Supplementary file 1 — Supplementary Material 1 [file 12882_2025_4657_MOESM1_ESM.docx]

**Manuscript**

**A narrative review of routine haematological and biochemical parameter monitoring in maintenance haemodialysis patients and comparison of clinical guidelines**

Harry H Luu^1,2^, Jade Ryan^1^, Nigel D Toussaint^1,3^

*^1^Department of Nephrology, The Royal Melbourne Hospital (RMH), Parkville, Australia;*

*^2^School of Medicine and Dentistry, Griffith University, Gold Coast, Australia;*

*^3^Department of Medicine (RMH), University of Melbourne, Parkville, Australia;*

**Supplementary Submissions**

Supplementary Table 1: Literature search strategy for narrative review on Ovid (MEDLINE and Embase).

| **Search Line Number** | **Searches** |
| --- | --- |
| 1 | exp “Renal Dialysis”/ |
| 2 | (“haemodialysis” OR “hemodialysis” OR “maintenance haemodialysis” OR “maintenance hemodialysis” OR “in-centre haemodialysis” OR “in-centre hemodialysis” OR “satellite haemodialysis” OR “satellite hemodialysis”) |
| 3 | 1 or 2 |
| 4 | exp “Clinical Laboratory Techniques”/ |
| 5 | (“pathology testing” OR “laboratory monitoring” OR “lab monitoring” OR “blood test” OR “blood tests” OR “blood testing” OR “routine testing” OR “routine blood tests” OR “routine labs” OR “biochemical monitoring”) |
| 6 | 4 or 5 |
| 7 | (“test frequency” OR “frequency” OR “interval” OR “monitoring schedule” OR “testing intervals” OR “test repetition” OR “over-testing” OR “under-testing” or “scheduled testing” OR “blood test frequency”) |
| 8 | exp “Health Care Quality, Access, and Evaluation"/ OR “Health Services Misuse” |
| 9 | (“appropriateness” OR “clinical utility” OR “value-based care” OR “overtesting” OR “test burden” OR “health care value” OR “unnecessary testing” OR “overtreatment” OR “low-value care” OR “cost-effectiveness”) |
| 10 | 8 or 9 |
| 11 | 3 AND 6 AND 7 AND 10 |
| 12 | exp “Peritoneal Dialysis”/ OR “Hemodiafiltration” |
| 13 | 11 NOT 12 |

Supplementary Figure 1: Flow Diagram for Narrative Review Search of Databases for Evidence of Frequency of Routine Pathology Testing and Clinical Guidelines Online Web-Search

**Identification of studies and clinical guidelines**

**via. database and other sources**

Records removed *before screening*:

Duplicate records removed (n = 78)

Records removed for other reasons (n = 2)

Records identified from: MEDLINE and Embase

Databases (n = 361)

Other sources such as Web Search (n = 26)*

**Identification**

Records screened

(n = 307)

Records excluded following title/abstract screening

(n = 249)

Reports not retrieved

(n = 2)

Full-text reports sought for retrieval

(n = 58)

**Screening**

Reports excluded: 35

Irrelevant outcome (n = 14)

Wrong study design (n = 9)

Conference abstract only

(n = 7)

etc.

Guideline lacked relevant monitoring details (n = 5)

Reports assessed for eligibility

(n = 56)

Studies included in review

(n = 8)

Clinical guidelines included in review (n = 13)

Total reports included in analysis (n = 21)**

**Included**

* The ‘other sources’ accounts for the international clinical guidelines that were primarily accessed via. online access.

** The total number of reports included as stated in the flow diagram does not account for literature sourced for background information that guided this narrative review.
